# Micro‐CT reconstruction reveals the colony pattern regulations of four dominant reef‐building corals

**DOI:** 10.1002/ece3.8308

**Published:** 2021-11-04

**Authors:** Yixin Li, Xin Liao, Kun Bi, Tingyu Han, Junyuan Chen, Jing Lu, Chunpeng He, Zuhong Lu

**Affiliations:** ^1^ State Key Laboratory of Bioelectronics School of Biological Science and Medical Engineering Southeast University Nanjing China; ^2^ Guangxi Key Lab of Mangrove Conservation and Utilization Guangxi Academy of Sciences Guangxi Mangrove Research Center Beihai China; ^3^ Nanjing Institute of Geology and Palaeontology Chinese Academy of Sciences Nanjing China; ^4^ Key Laboratory of Vertebrate Evolution and Human Origins of Chinese Academy of Sciences Institute of Vertebrate Paleontology and Paleoanthropology Chinese Academy of Sciences Beijing China; ^5^ CAS Center for Excellence in Life and Paleoenvironment Beijing China

**Keywords:** growth parameters, growth pattern, high‐resolution micro‐computed tomography, reconstruction, reef‐building coral

## Abstract

Colonies are the basic geometric building blocks of coral reefs. However, the forming regulations of both colonies and reefs are still not understood adequately. Therefore, in this study, we reconstructed 25 samples using high‐resolution micro‐computed tomography to investigate coral growth patterns and parameters. Our skeleton and canal reconstructions revealed the characteristics of different coral species, and we further visualized the growth axes and growth rings to understand the coral growth directions. We drew a skeleton grayscale map and calculated the coral skeleton void ratios to ascertain the skeletal diversity, devising a method to quantify coral growth. On the basis of the three‐dimensional (3D) reconstructions and growth parameters, we investigated the growth strategies of different coral species. This research increases the breadth of knowledge on how reef‐building corals grow their colonies, providing information on reef‐forming regulations. The data in this paper contain a large amount of coral growth information, which can be used in further research on reef‐forming patterns under different conditions. The method used in this study can also be applied to animals with porous skeletons.

## Graphical abstract

In this study, we use high‐resolution micro‐computed tomography to reconstruct 25 representative coral samples, indicating that there is a polyp network supported by the canal system within coral skeletons, which maintains coral growth. We visualized coral growth regulations to highlight the similarities and differences among different genera, and investigated different growth strategies by their skeleton‐forming patterns. Growth parameters of coral skeletons were obtained being on the gray scale of 3D reconstructions to reveal the mineralizing diversity among species.

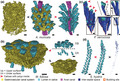

## 1 INTRODUCTION

*Acropora*, *Montipora*, *Pocillopora*, *Seriatopora*, *Cycloseris*, *Favites*, and *Porites* are widely distributed coral genera in the Indo‐Pacific region (Adjeroud et al., 2018; Prasetia et al., 2017) and the major offshore reef‐building corals in coral reefs (Costanza et al., 2014). Their diverse and varied growth forms provide a habitat and food for the many thousands of reef‐associated organisms (Ellison et al., 2005; Graham, 2014; Knowlton et al., 2010; Weis et al., 2008), making tropical reefs centers of biodiversity. Tropical coral reefs are under pressure from a range of local and global environmental stressors (Carpenter et al., 2008; Kleypas et al., 1999; Maynard et al., 2015), including global warming (Bellwood et al., 2004; De'ath et al., 2012; Jokiel & Coles, 1977) and ocean acidification (Mollica et al., 2018; Tambutté et al., 2015); environmental pollution (Kroon et al., 2019; Mantelatto et al., 2020) – for example, microplastics (Jeyasanta et al., 2020; McCormick et al., 2020; Patterson et al., 2020; Patti et al., 2020) and heavy‐metal contamination (Banc‐Prandi et al., 2020; Jafarabadi et al., 2020; Nour & Nouh, 2020; Yang et al., 2020); and overexploitation (Heard et al., 2020; Natt et al., 2017; Robinson et al., 2016), making the protection and ecological restoration of coral reefs critical. Studies on these predominant reef‐building corals are, therefore, gaining widespread traction (Adjeroud et al., 2009; Bramanti & Edmunds, 2016; Cunning et al., 2018; Edmunds et al., 2014; Kayal et al., 2012, 2015; Liang et al., 2017; Stolarski et al., 2016).

Conventional studies on reef‐building corals have concentrated on the physiology, ecology, statistics, and multi‐omics of corals, allowing us to understand the biological characteristics of coral polyps and symbiont zooxanthellae. At the heart of healthy reefs is the sustained formation of the calcium carbonate skeletons of many coral species; therefore, it is important to understand how corals grow their skeletons. The structure, distribution, volume ratio, and other useful patterns of the canals in coral skeletons are also important to understand, as they play key roles in coral growth (Li et al., 2021). Traditional biological analysis techniques, such as scanning electron microscopy (SEM) and grinding sections, necessitate a high workload and complex operability (Giuseppe et al., 2019; Odum & Odum, 1955). They provide limited evidence due to their non‐transparent coral skeletons, which restrict the direct observation of the structures and related parameters in coral colonies (Marfenin, 2015). In recent years, computed tomography (CT) has been applied to study reef‐building coral skeletons (Beuck et al., 2007; Gutiérrez‐Heredia et al., 2015; Knackstedt et al., 2006; Kruszyński et al., 2007; Kruszynski et al., 2006; Pinzón et al.,^2014^). With the development and popularization of high‐resolution micro‐computed tomography (HRCT), research on coral skeletons has revealed the morphological and internal structures of coral branchlets, and also investigated coral skeletal structures (Ivankina et al., 2020; Sentoku et al., 2015; Sentoku et al., 2015; Urushihara et al., 2016) and exogenous influences on coral skeletons, including water currents (Chindapol et al., 2013), the marine environment (Iwasaki et al., 2016), and ocean acidification (Enochs et al., 2016; Fordyce et al., 2020). However, there are various challenges in studying coral growth patterns regulation using CT sectional slices that are singly dependent on skeleton reconstruction. Consequently, we created a novel method of canal reconstruction, which used HRCT to study coral growth pattern regulation in *Pocillopora damicornis*, visualizing its calice networks, budding signals, polyp relationships, colony growth directions, and coral branching regulations (Li et al., 2020). Furthermore, to fill in current knowledge gaps, we expanded this method to determine the growth pattern in much more predominant reef‐building coral genera. Additionally, the basic growth parameters of coral colonies are difficult to quantify, and calculating skeleton values through HRCT reconstructions can mitigate this issue.

In this study, we used HRCT to systematically reconstruct the skeletons and canals of 25 representative *Acroporidae*, *Pocilloporidae*, *Fungiidae*, *Faviidae*, and *Poritidae* samples from the Indo‐Pacific region, including eleven species (*Acropora digitifera*, *Acropora muricata*, *Acropora millepora*, *Montipora foliosa*, *Montipora turgescens*, *Pocillopora verrucosa*, *Pocillopora meandrina*, *Seriatopora hystrix*, *Cycloseris vaughani*, *Favites speciosa*, and *Porites lutea*) in seven genera. Furthermore, we performed a series of experiments, including lumen reconstructions of the canal system in each colony, growth axis formation, growth ring visualization, grayscale gradient maps, and growth parameters quantification, to obtain the formation mechanism and related parameters for our colony samples. According to the canal reconstructions and measured growth parameters, we investigated different growth strategies of the major species to define how corals grow their skeletons and how skeletal growth varies among species. This work also provides some useful methods and extends our understanding of the digging growth information of coral colonies.

## 2 RESULTS

### 2.1 Canal reconstructions revealing coral growth patterns

**FIGURE 1:**
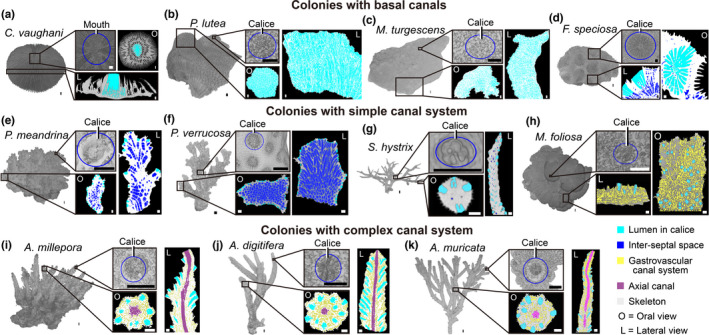
Coral skeleton structures and canal sites. (a–k) The reconstructions reveal both macroscopic and microscopic details of surface morphology, internal structural characters, and canal reconstructions in (a) *Cycloseris vaughani*, (b) *Porites lutea*, (c) *Montipora turgescens*, (d) *Favites speciosa*, (e) *Pocillopora meandrina*, (f) *Pocillopora verrucosa*, (g) *Seriatopora hystrix*, (h) *Montipora foliosa*, (i) *Acropora millepora*, (j) *Acropora digitifera*, (k) *Acropora muricata*. Colonies of species in a–d only have simple canal structures. *Cycloseris vaughani* is a monostomatous corallum, with only one large polyp in its mouth (1–2 cm in diameter). Calices of *P. lutea* and *M. turgescens* are exceedingly small (1 mm in diameter), closely packed and united by the walls (almost no space among calices), while adjacent polyps connect through their porous corallite walls and septa. Calices of *F. speciosa* are large (6–10 mm in diameter) and polygonal, and some inter‐septal space (1 mm in diameter) distribute around the calices. Colonies of species in e–h have simple canal systems. Calices of *P. meandrina* and *P. verrucosa* are small (0.5–1 mm in diameter), and their corallite walls touch each other. The inter‐septal spaces arrange neatly in columns. Calices of *S. hystrix* are round and slightly hooded (0.5 mm in diameter), while each lumen in calice corresponds to a small sphere inter‐septal space (0.1 mm in diameter) located in the center of the branch. The canal system in *M. foliosa* is different from *M. turgescens*, and the calices of *M. foliosa* (0.5–1 mm in diameter) are well separated by the coenosteum. Gastrovascular canal system and coenosteum connect the polyps in separated calices. *Acropora* species in i–k have complex canal system. Their calices (1 mm in diameter) are connected by the gastrovascular canal system, while axial canal is a unique structure in the canal system of these *Acropora* species. Scale bars: 1 mm

The 3D skeletal structures of 25 samples were reconstructed by HRCT at both the macroscopic and microscopic levels (Figure S1). The images in Figure 1 and Figure S1 show the complete forms of the original coral skeletons, including the surface morphology and internal structural characteristics, which facilitated an analysis of the coral colony pattern regulation. To obtain the coral growth information from skeletal structures, we reconstructed the canals inside the sample skeletons. The complex canal reconstructions can mainly be divided into three parts: the lumen in calices (where coral polyps live and grow), the inter‐septal space, and the gastrovascular canal system. The gastrovascular canal system connects all polyps in a colony, and the movement of fluids in it can transport materials to different parts of the colony, as required (Pearse & Muscatine, 1971). The axial canal is a unique canal within the axial corallite along the branches in an *Acropora* colony, and its extension reveals the branch growth directions. It belongs to the gastrovascular canal system, and there is also a polyp that lives at the tip of this canal (Li et al., 2021).

*Cycloseris vaughani* is a monostomatous corallum with a single central mouth. Spiny septa radiate from the mouth on the upper surface to the outer edge of the corallum, and the septal margins are armed with small blunt spines. Smooth costae radiate from the center of the solid under‐surface. These skeletons around the mouth form a calice‐like area for the growth of polyps. Since there is only one polyp in a colony, there is no canal system in *C. vaughani* (Figure 1a). *Porites lutea* are massive and encrusted with small calices (about 1 mm in diameter). Their colonies have slightly granular peritheca and porous corallite walls and septa. Adjacent corallites are compacted, with little coenosteum. The calices are closely connected by walls and arranged in a sector‐like formation (Figure 1b). The structure and configuration of calices in *M. turgescens* are similar to those in *P. lutea*, being slightly shorter in vertical length (Figure 1c). *Favites speciosa* has encrusting cerioid colonies with thick septa, elongated septal dentations, paliform lobes, columellae, and sturdy wall structures. Their septa are prominent and numerous, rising from the fossa, and the margins of the septa are spiny and dentated. The calices are polygonal, while the corallites are fused, with a common acute wall. Some inter‐septal spaces (1 mm in diameter) are arranged in columns and distributed around the calices. (Figure 1d). The colonies of *P. meandrina* and *P. verrucosa* are arborescent. The calices (with polyp inside) look like small shallow cups and are well separated by the coenosteum, covered with verrucae. The inter‐septal spaces (without polyps) are arranged in columns, following the calices where polyps used to live. Thin dissepiments isolate the inter‐septal spaces in one column, while thick thecas isolate the columns (Figure 1e,f). The calices of *S. hystrix* are round and slightly hooded, with minute tubercles and poorly developed septa within them. Each lumen in the calice corresponds to a small spherical inter‐septal space distributed near the center of the coral branches (Figure 1g). The canal system in *M. foliosa* differs from that of *M. turgescens*, and the calices of *M. foliosa* are well separated by the coenosteum. The gastrovascular canal system and coenosteum connects the polyps in separated calices. The coral skeleton is highly porous, while the corallite is small, with indistinct walls (Figure 1h). The three *Acropora* colonies have porous skeletal microstructures and small corallites, separated by a spongy coenosteum. The gastrovascular canal system, the lumen in the calice, and the axial canal in these *Acropora* colonies integrate the coral body like a circulatory system in higher animals (Figure 1i–k).

**FIGURE 2:**
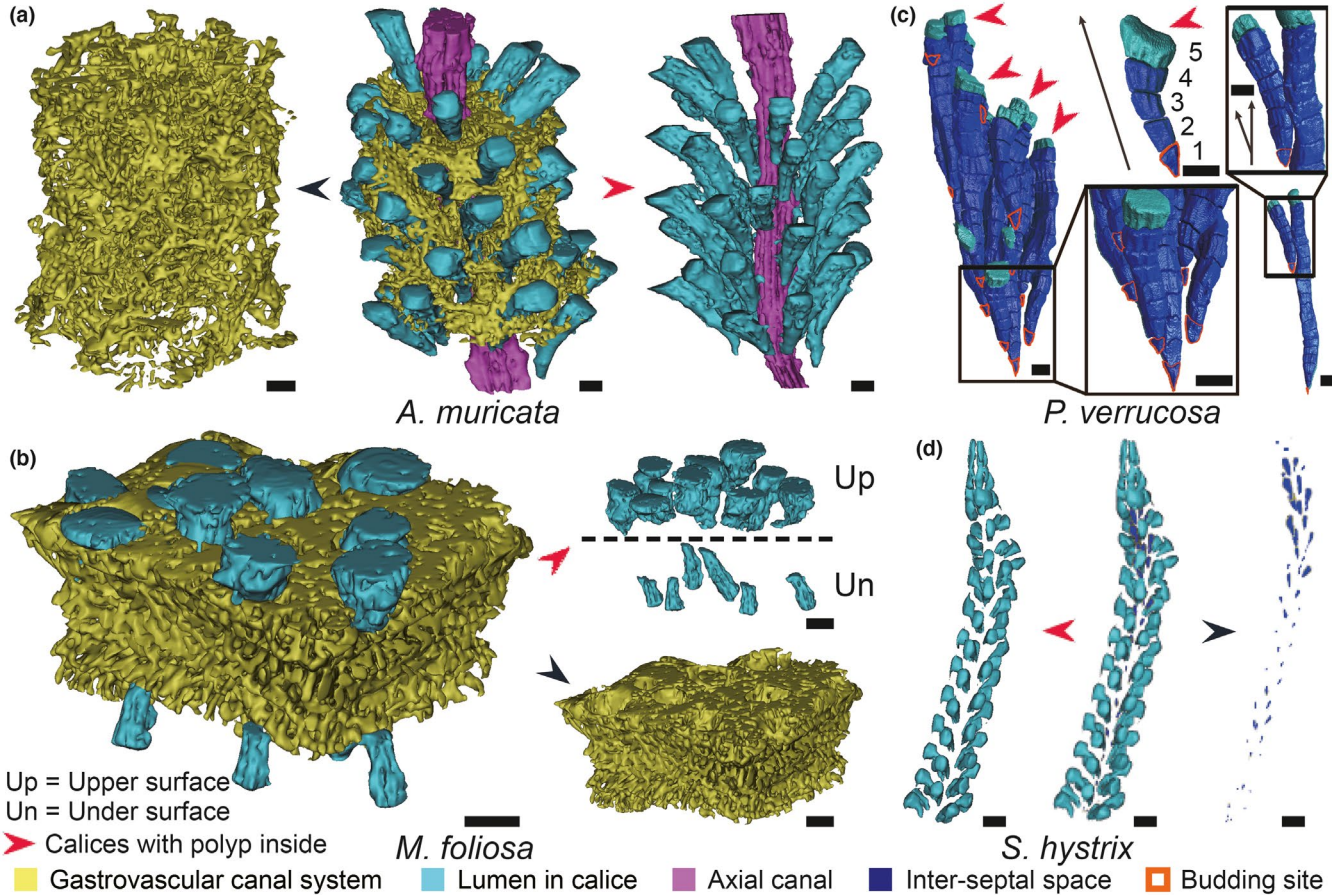
3D canal reconstructions of four typical reef‐building corals. (a) The gastrovascular canal system connects the axial canal and the lumen in calices (where polyps stay) in an *Acropora muricata* colony like a net. All these canals integrate the coral branch into an entirety like the circulatory system in higher animals. (b) In *Montipora foliosa*, the gastrovascular canal system forms a basic network, and the lumen in calices (with polyps inside) are regularly arranged in it. (c) Forming sequence and budding sites of the inter‐septal spaces and lumen in calices in *Pocillopora verrucosa* canal system. The newly budding polyp has a small inverted cone‐shaped inter‐septal space No. 1, and the following ones are grown into larger inverted truncated cones gradually (inter‐septal space No. 2–4 and lumen in calice No. 5). These inverted cone‐shaped spaces can be regarded as a kind of reminding information for polyp budding in the canal system of *P. verrucosa* colony, making it possible to capture the behaviors of all polyps in one colony of *P. verrucosa* by reconstruction. The budding sites can be accurately recorded regardless of the fate of certain individual polyp (Li et al., 2020). (d) The double‐layer canal system in *Seriatopora hystrix* is arranged spirally along with branch growth direction. Scale bars: 1 mm

The calices of coral samples in Figure 1e–k are more dispersed, while the coenosteum and more diverse canals in their colony connect adjacent calices. These coral species have more complex skeleton–canal structures and growth patterns. Therefore, we performed 3D canal reconstructions of the four species (*A. muricata*, *M. foliosa*, *P. verrucosa*, and *S. hystrix*) with their representative canal systems to reveal how these internal structures under coral skeletons integrate all polyps in one colony during coral growth (Figure 2).

In every *A. muricata* branch, the distance between adjacent calices is similar, and the lumen in calices encircles the axial canals along the growth direction. The gastrovascular canal system wraps all the calices like a thick net, connecting them around the axial canal. The distances from the bottom of each lumen in the calices to the axial canal are also similar, and the gastrovascular canal system links them together (Figure 2a). The gastrovascular canal system in *M. foliosa* forms a basic network where the calices are inserted regularly and arranged in order. Because sunlight can shine only on upper surfaces and penetrate the edges of the under‐surfaces, calices are widely distributed in the upper surfaces, with a small number in the under‐surface margins. On the edge of an *M. foliosa* colony, the upper and under calices are separated by the gastrovascular canal system. The calices on the under‐surfaces are smaller and more sparsely distributed than those on the upper surfaces (Figure 2b). In a *P. verrucosa* colony, polyps keep forming new calices during the coral growth process. Once a new calice is built, the polyp leaves its old calice and enters the new one (Li et al., 2020). Every newly budded polyp forms a small calice, with inverted cone‐shaped lumen inside (e.g., inter‐septal space No. 1 in Figure 2c), while the later‐formed calices contain larger inverted truncated cone spaces (e.g., inter‐septal space No. 2–4 and the lumen in calice No. 5 in Figure 2c). These inverted cone‐shaped spaces can be regarded as a reminder for polyps budding in the canal system of a *P. verrucosa* colony, making it possible to capture the behaviors of all polyps in one corallum of *P. verrucosa* by canal reconstruction, whereby budding sites can be accurately recorded regardless of the fate of the individual polyps (Figure 2c). In *S. hystrix* colonies, calices contain cup‐shaped lumens with a larger volume, each corresponding to their smaller droplet‐like inter‐septal spaces. There are no obvious connections between the lumens in calices and inter‐septal spaces, and both canals are arranged spirally along the growth direction of the coral branches. The inter‐septal spaces generally appear in groups, arranged in the form of three to four independent droplet‐like canals that are gathered together. For each group of internal canals, there is a group of surrounding calices (with polyps inside) of a similar quantity (Figure 2d).

It has been found that all polyps in one *P. verrucosa* colony participate in multiple biological processes through the canal system, including the budding, branching, mineralization, and movement trajectory of polyps (Li et al., 2020). In *P. verrucosa*, the polyp network is supported by the coenosteum and the network of inter‐septal spaces that connects all polyps in one colony, which is universal in *Pocillopora* (Figure 1e,f, Figure 2c). In *Acropora* and *Montipora*, the polyp network is more complex because more canal types are involved. The gastrovascular canal system connects all polyps in a holistic network to collaboratively perform biological processes in a single coral colony.

### 2.2 Growth axis and ring visualization based on 3D reconstruction

**FIGURE 3:**
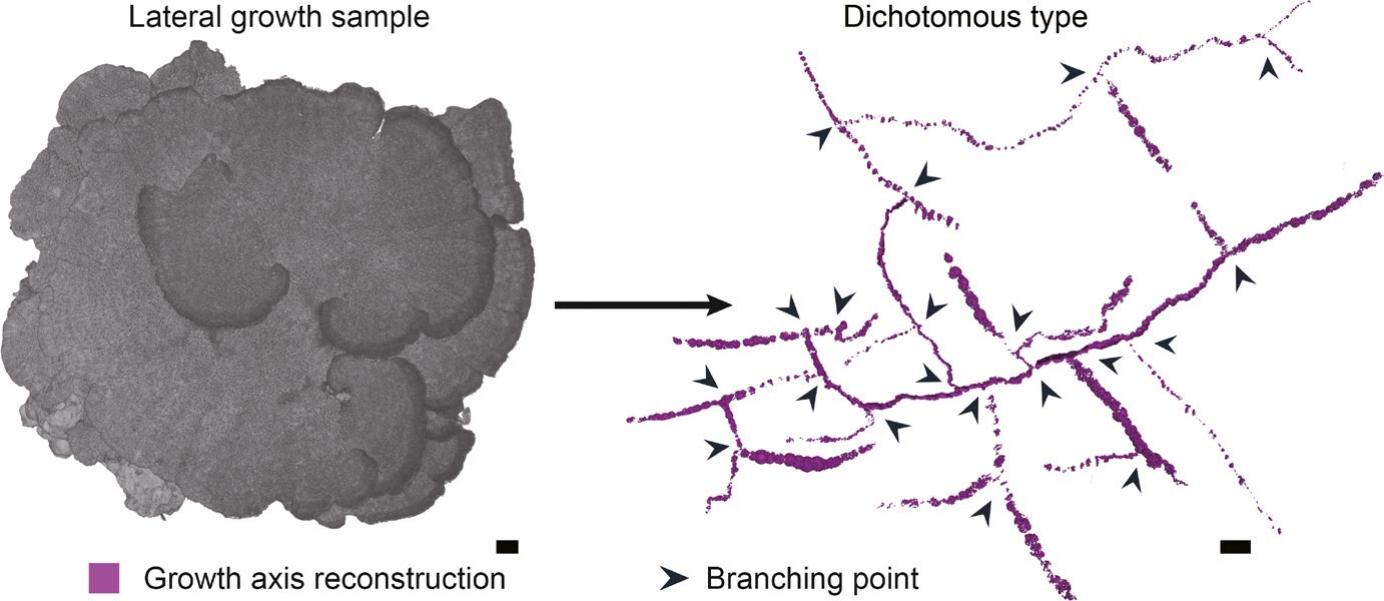
Growth axis reconstruction of *Montipora foliosa*. The growth axis reconstruction of our lateral growth sample, *M. foliosa*, was dichotomous type. The growth axis extends to the perimeter from the center of *M. foliosa* colony, and foliose‐shaped skeletons are formed along the direction of the growth axis. The growth axis and coral branch will both split into two parts at the branching point, while new branches will keep growing along the direction of the new growth axis. Thus, we call it the “dichotomous type”. This net‐like growth axis ensures that this lateral growth species can maximize the use of light resources (Li et al., 2020). Scale bars: 1 cm

The canal reconstruction revealed that coral colonies follow specific growth axes (Li et al., 2020); this can accurately reflect how coral reefs are formed. To illustrate the coral growth direction at the level of polyp proliferation and skeletal dynamic accretion, we visualized the growth axes of various colonies through the 3D reconstruction of canal systems. This regulation cannot be observed directly in coral skeletons and their primary morphology (Figure 3 and Figure S2). Growth direction reconstruction in corals following axial growth, such as *A. muricata* and *S. hystrix*, can be simply accomplished using basic morphological information. *A. muricata* growth follows its axial canal (Figure 2A and Figure S2A), and the gastrovascular canal system in *S. hystrix* plays a similar role (Figure 2d). However, the growth axes following radial‐ or lateral‐growth coral, such as in *P. verrucosa* and *M. foliosa*, are not obvious. To analyze the growth axes of radial‐growth coral, for instance, *P. verrucosa*, we reconstructed the calices from the branching point to the uppermost tips of each branchlet (Figure S2B). *M. foliosa* is a typical lateral‐growth coral lacking an axis canal, so we improved our former reconstruction method to explore its growth axis (Figure S2C,D). An *M. foliosa* branch expands outward in a foliose shape, and the canal reconstruction from the proximal bifurcating point to the distal edge revealed that the growth direction forms a foliose‐shaped branch. Using this method, we reconstructed the entire growth axis of an *M. foliosa* colony, which reflected a regular dichotomous type (Figure 3). The reconstruction results show that the investigated growth axes of reef‐building corals can be divided into three types: dichotomous, polytomous, and divergent (Figure S2E). The dichotomous growth axis (*P. verrucosa*, *A. digitifera*, and *M. foliosa*) bifurcates at all forks, whether on the main branch or branchlets. The polytomous growth axis (*A. muricata* and *S. hystrix*) divides into two or more parts during the branching process. The divergent growth axis (*A. millepora* and *P. meandrina*) has no main branches.

Growth rings reflect the details of coral growth and were obtained through the 3D reconstructions of canal systems and growth axes. These rings can divide one coral branch into multiple layers, showing the entire process of the growth pattern (Figure S3). In the *M. foliosa* sample, the initial growth ring was quite small, which then proliferated along with the growth axis through the canal system (Figure S3A). The mature growth rings of *A. digitifera* and *S. hystrix* maintained a similar volume throughout branch growth (Figure S3B,D). In *P. verrucosa*, the volumes of the growth rings varied and depended on temporal ecological factors (Figure S3C).

In summary, the 3D reconstructions showed that the coral growth patterns were regulated by a hierarchy of canal systems, growth axes, and growth rings, while the canal system was its basic foundation. The canals within reef‐building coral colonies have regulator distributions, and the colony pattern or growth model can be traced by investigating this kind of regulation.

### 2.3 Coral growth parameter analyses based on 3D reconstruction

**FIGURE 4:**
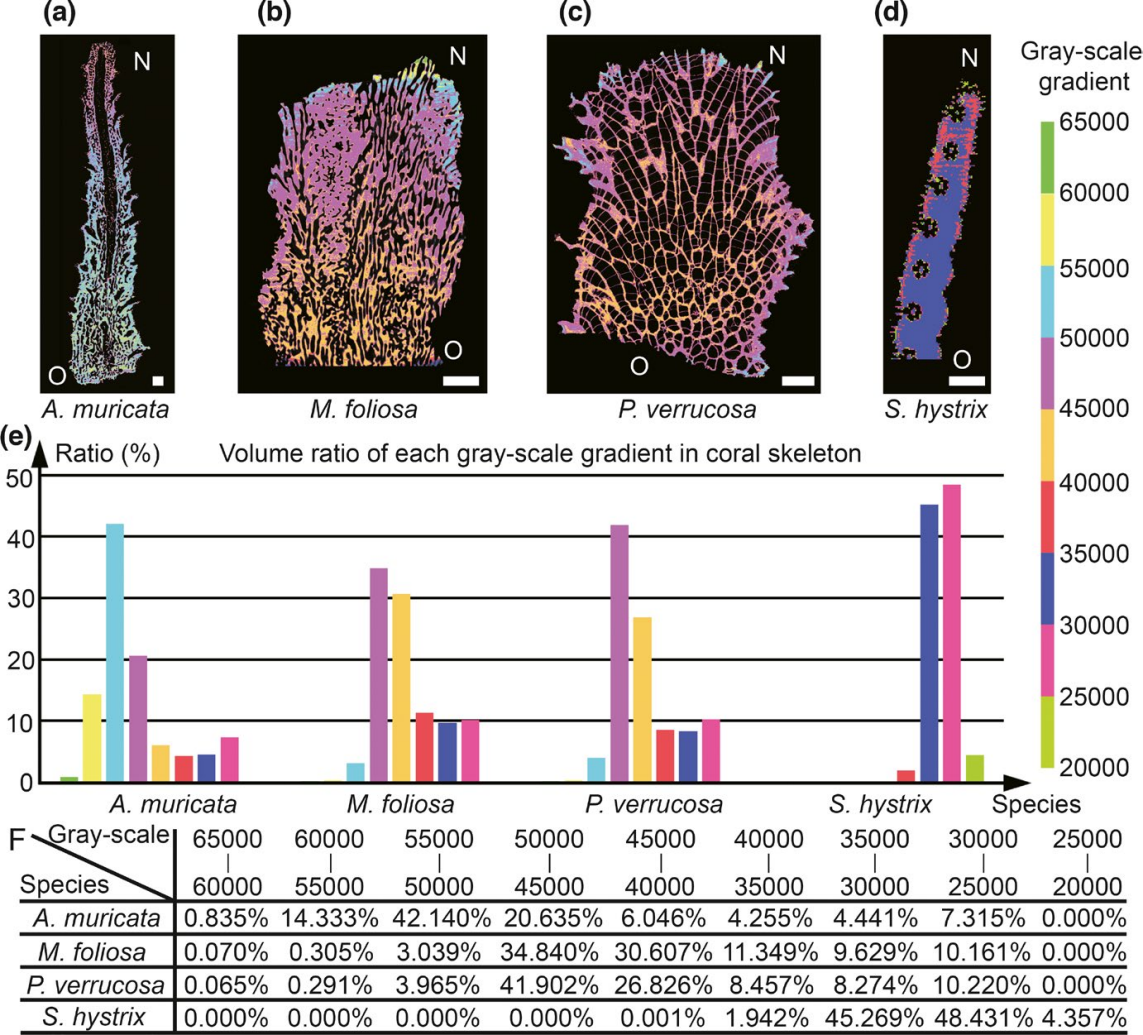
Grayscale gradient map reveals density distribution of coral skeletons. (a–d) The HRCT results can divide all coral skeleton reconstructions into nine parts with a gradient of 5000 gray scales within the range of 20,000–65,000 and color them to show the different distribution of skeleton density. O = Old, N = New. (e, f) Volume ratio of each grayscale gradient in coral skeleton of the four species. The value in Table f corresponds to each colored rectangle in the histogram e. Scale bars: 2 mm

**FIGURE 5:**
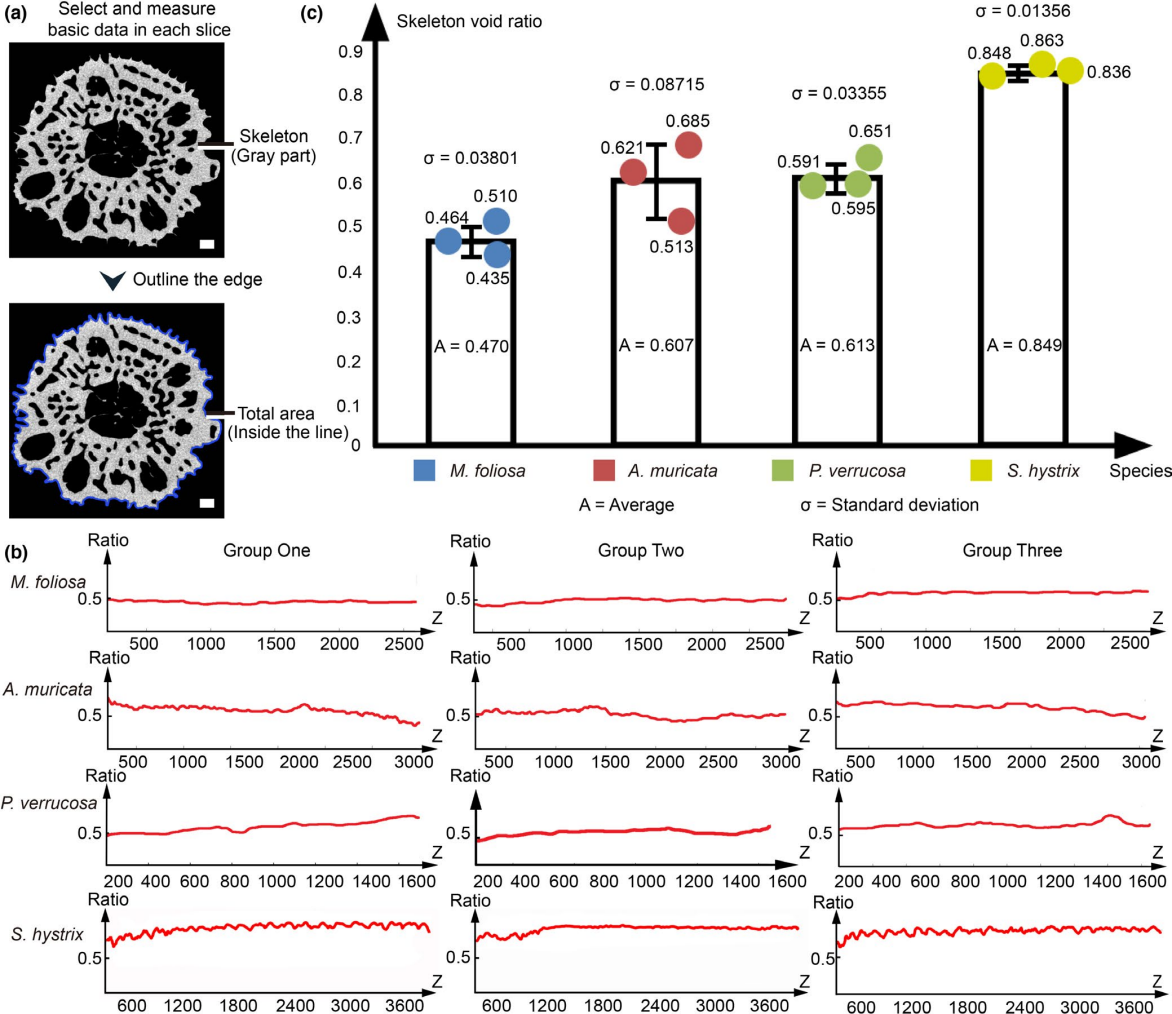
Growth parameter analysis through colony reconstruction. (a) By outlining the edges of the reconstructed branches in the slices, we calculated the total acreage of skeleton and canals in each slice. The length of edge line shows the perimeter of coral branch in each slice. (b) We defined the direction of growth axis as *Z*‐axis in the 3D coordinate system of coral reconstruction, and the *X*–*Y* plane is the radial cross‐section perpendicular to *Z*‐axis. The skeleton ratio along *Z*‐axis of coral colony was shown in the line charts. (c) The skeleton ratio of the coral samples in *Montipora foliosa*, *Acropora muricata*, *Pocillopora verrucosa*, and *Seriatopora hystrix*. Each coral genera contain three samples. Standard deviation is shown as the value of σ in the histogram. Scale bars: 1 mm

According to our 3D reconstructions, the growth parameters – including skeletal density and the skeleton void ratio – can be obtained, facilitating the analysis of coral growth regulation (Figure 4, Figure 5, and Table 1). The grayscale of a 3D CT reconstruction reflects the skeletal density, and samples with higher grayscale values have higher skeletal densities (Chugh et al., 2016) (Figure 4a–d). The volume ratio of each grayscale gradient in the coral skeleton of the samples can be calculated to quantify the mineralizing differences (Figure 4e,f). The grayscale range of the *A. muricata* skeleton (Figure 4a,e,f) is approximately 45,000–60,000, and that of a new mineralized skeleton (40,000–50,000) is lower than that of an old skeleton (50,000–65,000). The grayscale range of the *M. foliosa* skeleton (Figure 4b,e,f) is approximately 40,000–50,000, and that of a new mineralized skeleton (50,000–65,000) is higher than that of an old skeleton (25,000–50,000). The grayscale range of a *P. verrucosa* skeleton (Figure 4c,e,f) is approximately 40,000–50,000, and that of a new mineralized skeleton (50,000–65,000) is higher than that of an old skeleton (30,000–45,000). The grayscale range of dissepiment is approximately 25,000–40,000. The grayscale range of an *S. hystrix* skeleton (Figure 4d,e,f) is approximately 25,000–35,000, and that of a new mineralized skeleton (20,000–30,000) is lower than that of an old skeleton (30,000–35,000). The results also show that different areas in one coral colony have different skeletal densities, and statistics suggest that the skeletal densities among different species are distinct and in the descending order of *A. muricata*, *P. verrucosa*, *M. foliosa*, and *S. hystrix* (Table 1). In *A. muricata* and *S. hystrix*, the density of new growth skeletons is lower than that of old ones (Figure 4a,d and Table 1). However, the case for *M. foliosa* and *P. verrucosa* is the opposite (Figure 4b,c and Table 1). Coral colonies are filled with canals, and it is difficult to precisely measure coral skeleton void ratios (the proportion of skeletal material to the total volume) (Naumann et al., 2009). To mitigate this issue, we designed a method to calculate coral skeleton void ratios according to micro‐CT reconstructions and grayscale analysis (Figure 5a, Methods and Materials). Through related constructions and grayscale data, we could calculate both the skeleton void ratio at any slice (Figure 5b) and the total skeleton void ratio (Figure 5c). The results show that the average skeleton ratios of *Montipora capricornis*, *A. muricata*, *P. verrucosa*, and *S. hystrix* are 47.0%, 60.7%, 61.3%, and 84.9%, respectively (Figure 5c). These data indicate that the coral mineralization values differ significantly between species, which play an important role in the diverse growth patterns and survival strategies of coral.

**TABLE 1:** Grayscale ranges for coral samples

Coral sample	Approximate grayscale range	New skeleton	Old skeleton
*Acropora muricata*	45,000–60,000	40,000–50,000	50,000–65,000
*Montipora foliosa*	40,000–50,000	50,000–65,000	25,000–50,000
*Pocillopora verrucosa*	40,000–50,000	50,000–65,000	30,000–45,000
*Seriatopora hystrix*	25,000–35,000	20,000–30,000	30,000–35,000

Note

Higher gray scale indicates higher skeletal density.

## 3 DISCUSSION

### 3.1 Reef colony forming strategy comparison based on 3D reconstructions

The canal system in the skeleton of a colony is the basic foundation for coral growth (Figure 2), while the type and direction of the growth axis determine the branching pattern and growth direction of the colony, respectively (Figure 3, Figure S2). According to the canal reconstructions in this study, we investigated different growth strategies of four representative species to define how corals grow their skeleton and how skeletal growth varies among species.

The corals of each species have their own unique reef forming strategies. The density of new skeletons in *M. foliosa* and *P. verrucosa* is higher than that of old ones, while the patterns in *A. muricata* and *S. hystrix* are just the opposite (Figure 4, Table 1). *Montipora foliosa* is a lateral‐growth coral, with thinner skeletons at colony edge that are susceptible to physical damage. Therefore, the density of new skeletons formed at the colony edge is higher, ensuring its mechanical strength (Figure 4b). For *P. verrucosa*, a radial‐growth coral with fragile dissepiments, the new skeletons with high densities are mineralized on the surface of the colony to protect inner structures (Figure 4C). Further, axial‐growth corals – such as *A. muricata* and *S. hystrix* – form new skeletons with lower densities, because the protection of entire colony is not dependent on the growth area at the branch tip (Figure 4a,d).

*Montipora foliosa* colonies have the lowest skeleton ratios (47.0%) of the four major species (Figure 5c). This is primarily because its horizontal growth pattern is less affected by sea waves, while its high skeleton density (Figure 4e,f) also helps maintain the mechanical strength of the colony. Thus, the rigidity and strength of the coral colony can be sacrificed to speed up the expansion of its growth rings, increasing its capacity for occupying ecological niches. The other three longitudinally growing genera require higher skeletal ratios to maintain their strength. As *S. hystrix* colonies are normally found in habitats exposed to wave action, a higher skeleton ratio of 84.9% (highest of these four species) is needed to withstand the impact of waves (Figure 5c). Furthermore, the calices of *S. hystrix* are rather long and thin (Figure 2d), offering better protection to polyps during the day. The skeletal ratio of *A. muricata* colonies (60.7%) is lower than that of *S. hystrix* (Figure 5c); however, their higher skeleton density and thicker branches ensure sufficient hardness (Figure 4e,f), and the porous skeletal microstructure – with the most complex canal system of these species – allows for more strength for weight bearing (Figure 2). Most skeletons in *P. verrucosa*, which occur from exposed reef fronts to protect fringing reefs, have higher density (Figure 4e,f). However, the surface skeletons of *P. verrucosa* colonies are also much thicker than those of the other three species (Figure 1). The skeletal ratio of *P. verrucosa* is only 61.3%, which similar to that of *A. muricata* (Figure 4e,f), because the thin dissepiments and multiple calices inside the colonies lower the average ratio (Figure 4c). These coral growth strategies are closely related to the habitats of each species and reveal how coral skeletons grow, providing a theoretical basis for the further protection and restoration of tropical coral reefs.

We believe that the pattern of coral growth rings is related to the growth axis and coenosteum. The growth rate of polyps that share coenosteums in a colony is synchronized, and the polyps mineralize their coenosteums along the direction of the growth axis. As the environment will affect the morphology and density of skeletons mineralized at different time periods, there are multiple ‘rings’ of different skeletons in one colony, each corresponding to a specific time period. The growth rings are more specific in species with solid coenosteums, like *P. verrucosa*. In *P. verrucosa*, different environmental conditions – such as light – affect the canal and skeletal structures of each growth ring within the colony (Li et al., 2020). The related growth parameters can be obtained by reconstructing and measuring the inter‐septal spaces in each growth ring, and performing cluster analysis to infer the light conditions received by the colony when this part of the skeleton is formed. The skeletal density of the *P. lutea* colony is found to be related to seawater temperature, rainfall, and sunshine hours, while the density of skeletons in each growth ring fluctuates quasi‐12‐month periodically (Shi et al., 2004). Therefore, the grayscale gradient map can be compared to the growth ring reconstructions to study the monthly and yearly influence of the marine environment on coral skeletons. To quantify the specific correlations among the growth ring, coral skeleton, and environmental factors, more experimentation and comparisons are necessary.

In prior studies, researchers found that the atolls in the South China Sea have similar features that indicate forming regulation by basic coral colonies (Liu et al., 2019). We found that the pattern regulation of the *M. foliosa* colony is similar to that of atolls (Figure S3A). The horizontally extending arc‐shaped growth rings in *M. foliosa* colonies are similar to the atolls in the South China Sea. This self‐similarity feature reveals the connection between a single colony and the entire reef, providing a model for studying the forming regulation of macro‐scale reefs according to micro‐scale colony reconstructions.

### 3.2 Skeleton–canal reconstruction as a method for studying animals with porous skeletons

In this study, we determined the growth pattern regulation of major reef‐building coral species through 3D reconstructions of canals and skeletons using HRCT. These canal systems and skeleton data support further studies on reef‐building corals and improve upon the knowledge base of coral biology, including structural features, growth regulation, mineralization traits, and polyp networks (Li et al., 2020). At the surface of a reef‐building coral branch, all polyps are interconnected like a vertebrate's dermal system (Dubininkas, 2017) by tissue, making up an apparent polyp network. Here, we found that there is another type of polyp network supported by the canal system within a skeletal system that maintains coral growth processes.

The canal reconstructions allowed us to obtain the mechanism behind coral growth, budding, branching, and mineralizing, which helped visualize the growth process, summarize growth strategies, and ascertain growth models (Figures 1, 2, 3). Following the growth direction shown in the canal systems, we reconstructed the growth axis of coral colonies, particularly *M. foliosa*, which seems to grow irregularly. This research could provide a solid basis for coral classification (Figure S2). To obtain a deep understanding of coral growth patterns at the monthly, seasonal, and yearly time scales, reconstructing the growth pattern hierarchy of canal system, growth axes, and growth rings is of great importance (Figure S2E, Figure S3). Additionally, we reconstructed coral skeletons through HRCT to draw grayscale gradient maps, revealing the density differences of coral skeletons in each colony and quantifying the volume ratio of skeletons within specific density intervals, which reflected the differentiated mineralization strategies (Figure 4). On this basis, we calculated the reconstructed skeletons layer‐by‐layer to obtain the growth parameters, including coral mineralization, surface areas, and skeleton ratios, which help quantify coral growth (Figure 5). The related data contain a large amount of information on coral growth, which can be used for further research on coral growth patterns in different conditions or time periods.

A large number of animals have skeletons with complex canal systems, similar to reef‐building corals. In a manner mimicking the annual rings of a tree, these skeletons and canal systems contain significant amounts of growth information, including calcification strategies and growth patterns. Based on the reconstructions of these skeletons and canals, we can easily obtain growth parameters, summarize growth regulations, and visualize the growth process, laying the foundation for the construction of animal models. Thus, we believe that the above‐mentioned skeleton–canal reconstruction method is not only suitable for studying most coral species, but can also be applied to numerous animals with porous skeletons.

## 4 METHODS AND MATERIALS

### 4.1 Sample collection

The *A. muricata*, *A. millepora*, *M. foliosa*, *P. verrucosa*, *P. meandrina*, and *C. vaughani* samples were collected from the Xisha Islands; the *A. digitifera*, *S. hystrix*, *F. speciosa*, and *M. turgescens* samples were collected from the Nansha Islands; the *P. lutea* sample was collected from the Weizhou Islands (Figure S4). All samples, which occurred in large arborescent colonies forming thickets, were found in tropical shallow reefs of marine neritic, from depths of about 5–10 m. The daily mean temperature was between 23.2 and 29.2°C. The coral samples were kept whole and housed in our laboratory coral tank, where all conditions were simulated to reflect those of their habitat in the South China Sea. These samples were kept in the tank for about 1–3 months before the HRCT test. Among these samples, 12 of them are *Acroporidae*, with six *Acropora* samples (one 15 * 15 * 25 cm *A. digitifera* colony, one 20 * 20 * 25 cm *A. muricata* colony, one 10 * 10 * 10 cm *A. millepora* colony, and three *A. muricata* branches with the size around 1 * 1 * 4 cm) and six *Montipora* samples (two 30 * 25 * 15 cm *M. foliosa* colonies, one 10 * 10 * 1 cm *M. turgescens* colony, and three *M. foliosa* branches with the size around 4 * 4 * 1 cm). Ten samples are *Pocilloporidae*, including six *Pocillopora* samples (two *P. verrucosa* colonies, including a 20 * 20 * 15 cm bigger sample and a 6 * 6 * 8 cm smaller sample, one 15 * 15 * 10 cm *P. meandrina* colony and three *P. verrucosa* branches with the size around 2.5 * 1.5 * 3 cm) and four *Seriatopora* sample (one 10 * 5 * 5 cm *S. hystrix* colony and three *S. hystrix* branches with the size around 0.5 * 0.5 * 3 cm). The other three samples include one 6 * 6 * 3 cm *C. vaughani* colony, one 4 * 4 * 3 cm *Favites speciosa* colony, and one 8 * 8 * 6 cm *Porites lutea* colony.

### 4.2 Coral culture system

Our coral samples were cultured with the laboratory auto‐calibration balance system in a standard Red Sea^®^ tank (redsea575, Red Sea Aquatics Ltd.), following the Berlin method. The temperature was kept at 25 °C, and the salinity (Red Sea Aquatics Ltd.) was 1.025. The culture system was maintained using a Protein Skimmer (regal250s, Honya Co. Ltd.), a water chiller (tk1000, TECO Ltd.), three coral lamps (AI^®^, Red Sea Aquatics Ltd.), two wave devices (VorTechTM MP40, EcoTech Marine Ltd.), and a calcium reactor (Calreact 200, Honya Co. Ltd.).

Around 20 kg of live rocks, which were also collected from the South China Sea, was placed in the coral tank. These live rocks provided the structure of the growth environment and some necessary microorganisms. We also added minerals to the tank weekly, including Mg, Ca, KH, K, I, and Fe.

### 4.3 HRCT test

We analyzed 25 samples (six *Acropora* samples, six *Montipora* samples, six *Pocillopora* samples, four *Seriatopora* samples, one *Cycloseris* sample, one *Favites* sample, and one *Porites* sample) from the South China Sea using three‐dimensional models constructed with the 230 kV latest‐generation X‐ray microfocus computed tomography system (Phoenix v|tome|x m, General Electric (GE)) at Yinghua NDT, Shanghai, China. Two‐dimensional image reconstructions of each specimen from matrices of scan slices were assembled using proprietary software from GE. The relevant parameters are shown in Table 2.

**TABLE 2:** Parameters of the HRCT tests

Samples	Voltage	Current	Voxel size	Timing	Number of images	Image width	Image height
*Acropora digitifera* colony	150 KV	180 μA	49 μm	1 s	1800	3990 pixels	4000 pixels
*Acropora muricata* colony	130 KV	170 μA	25 μm	1 s	1500	3990 pixels	4000 pixels
*Acropora millepora* colony	120 KV	90 μA	37 μm	1 s	1800	3990 pixels	4000 pixels
*Acropora muricata* branch one	140 KV	70 μA	10 μm	500 ms	1500	2500 pixels	4000 pixels
*Acropora muricata* branch two	120 KV	115 μA	13 μm	500 ms	2000	3990 pixels	4000 pixels
*Acropora muricata* branch three	120 KV	90 μA	9 μm	500 ms	1700	2300 pixels	4000 pixels
*Montipora foliosa* colony one	190 KV	250 μA	49 μm	1 s	1400	3990 pixels	4000 pixels
*Montipora foliosa* colony two	220 KV	210 μA	47 μm	1 s	1500	3990 pixels	4000 pixels
*Montipora turgescens* colony	160 KV	220 μA	37 μm	500 ms	1500	3000 pixels	4000 pixels
*Montipora foliosa* branch one	120 KV	90 μA	14 μm	1 s	1400	3990 pixels	4000 pixels
*Montipora foliosa* branch two	170 KV	100 μA	15 μm	500 ms	1600	2024 pixels	2024 pixels
*Montipora foliosa* branch three	200 KV	100 μA	18 μm	500 ms	1600	2024 pixels	2024 pixels
*Pocillopora verrucosa* colony one	210 KV	205 μA	87 μm	1 s	2000	2000 pixels	2000 pixels
*Pocillopora verrucosa* colony two	220 KV	120 μA	12 μm	1 s	1500	3990 pixels	4000 pixels
*Pocillopora meandrina* colony	130 KV	170 μA	25 μm	1 s	1500	3990 pixels	4000 pixels
*Pocillopora verrucosa* branch one	220 KV	120 μA	18 μm	1 s	2000	2000 pixels	2000 pixels
*Pocillopora verrucosa* branch two	180 KV	130 μA	16 μm	1 s	2000	2000 pixels	2000 pixels
*Pocillopora verrucosa* branch three	220 KV	120 μA	7 μm	1 s	1500	3990 pixels	4000 pixels
*Seriatopora hystrix* colony	160 KV	140 μA	22 μm	500 ms	1800	3990 pixels	4000 pixels
*Seriatopora hystrix* branch one	200 KV	110 μA	6 μm	500 ms	1400	2200 pixels	4000 pixels
*Seriatopora hystrix* branch two	200 KV	110 μA	5 μm	500 ms	1400	2200 pixels	4000 pixels
*Seriatopora hystrix* branch three	200 KV	110 μA	6 μm	500 ms	1400	2200 pixels	4000 pixels
*Porites lutea* colony	160 KV	230 μA	20 μm	334 ms	1500	3990 pixels	4000 pixels
*Favites speciosa* colony	160 KV	230 μA	19 μm	334 ms	1500	3990 pixels	4000 pixels
*Cycloseris vaughani* colony	120 KV	100 μA	13 μm	1 s	1600	3990 pixels	4000 pixels

### 4.4 Internal canal reconstruction

Slice data derived from the scans were then analyzed and manipulated using VG software. The 3D reconstructions were created in Mimics (v20.0) software and VG Studio Max (v3.3.0), following the method as previously described (Li et al., 2020). The images of the reconstructions were exported from Mimics and VG Studio Max and finalized in Adobe Photoshop CC 2019 and Adobe Illustrator CC 2019.

### 4.5 Coral‐growth quantitative analysis

We randomly selected three coral branches from colony reconstruction results of *M. foliosa*, *A. muricata*, *P. verrucosa*, and *S. hystrix*. We defined the direction of growth axis as *Z*‐axis in the 3D coordinate system of coral reconstruction, and the *X*–*Y* plane is the radial cross‐section perpendicular to *Z*‐axis. Then, we exported each reconstructed branch as a set of slices perpendicular to the *Z*‐axis (Figure 5a). The thickness of each slice was one pixel while the number of slices depended on the length of each coral branch. The specific value of one pixel depends on the detector resolution of the HRCT reconstruction data shown in the method (e.g., the thickness of one slice in *A. muricata* branch one is 10 μm). The white part is the acreage of corallite in each slice. By outlining the edges of the reconstructed branches in the slices (Figure 5a), we revealed the total acreage of skeleton and canals in each slice, which represents the total niche space of each slice. The length of edge line shows the perimeter of coral branch in each slice (Appendix Code).

We calculate the proportion of the skeleton acreage to the total branch acreage in every slice to obtain a curve of the skeleton ratio of each slice along the *Z*‐axis. Then, we calculate the integral of this curve for the skeleton ratio of coral branch. We select two points on the branch arbitrarily and find their positions on the *Z*‐axis. Then, we calculate the integral of the skeleton area curve between them for the coral mineralization of this area. Meanwhile, calculating the integral of the coral perimeter curve can reveal the surface area of the coral branch except the subface. As all calices with polyps of *M. foliosa*, *A. muricata*, *P. verrucosa*, and *S. hystrix* are found in the surface layer with exception of the subface, the calculation result above can be regarded as potential light absorption area of these coral colonies.

## CONFLICT OF INTEREST

The authors declare no competing interests.

## AUTHOR CONTRIBUTIONS

**Yixin Li:** Conceptualization (equal); Data curation (lead); Formal analysis (lead); Investigation (lead); Methodology (lead); Resources (equal); Software (lead); Validation (lead); Visualization (lead); Writing‐original draft (lead); Writing‐review & editing (lead). **Xin Liao:** Resources (equal); Writing‐review & editing (supporting). **Kun Bi:** Formal analysis (supporting). **Tingyu Han:** Data curation (supporting). **Junyuan Chen:** Writing‐review & editing (supporting). **Jing Lu:** Software (supporting); Writing‐review & editing (supporting). **Chunpeng He:** Conceptualization (equal); Funding acquisition (equal); Resources (equal); Supervision (equal); Writing‐review & editing (supporting). **Zuhong Lu:** Conceptualization (equal); Funding acquisition (equal); Resources (equal); Supervision (equal); Writing‐review & editing (supporting).

### OPEN RESEARCH BADGES

This article has earned an Open Data Badge for making publicly available the digitally‐shareable data necessary to reproduce the reported results. The data is available at *Acropora* colony 1 (*Acropora digitifera*): https://doi.org/10.5061/dryad.cc2fqz65g: https://datadryad.org/stash/share/JPGMRKQbQ8_87uWjAKvFYIdxZXXXS94Hm8dpDAO5Np4. *Acropora* colony 2 (*Acropora muricata*): https://doi.org/10.5061/dryad.sf7m0cg5q: https://datadryad.org/stash/share/s0FVMlF4KcOizXGbVJSBYL8TaJKUd0R0meZ4ac0f2wg. *Acropora* colony 3 (*Acropora millepora*): https://doi.org/10.5061/dryad.3r2280gfp: https://datadryad.org/stash/share/hX1oJdlviBy3ZvrDU_MHUzs3KyC4Tj4rKJnDMUtGYqU. *Acropora* branch 1 (*Acropora muricata*): https://doi.org/10.5061/dryad.bk3j9kdb8: https://datadryad.org/stash/share/8slxGygK_qcnQ2FgmSzAD5MjNjnp8U‐Gz8tnB64Ttbc. *Acropora* branch 2 (*Acropora muricata*): https://doi.org/10.5061/dryad.0cfxpnw1t: https://datadryad.org/stash/share/Hkzyc9vigW1BvdXi7xLUZNcpDl0RQ1X1NiVxErc4Axg. *Acropora* branch 3 (*Acropora muricata*): https://doi.org/10.5061/dryad.vt4b8gtrm: https://datadryad.org/stash/share/‐PxLGIBKoPLzjNqzhWX8xS1jEoEbzhwa0ixhEQPb9hc. *Montipora* colony 1 (*Montipora foliosa*): https://doi.org/10.5061/dryad.08kprr525: https://datadryad.org/stash/share/nUY3EePPILdaehpbHw4XUOccxVZ3DwSSgNSoRuV8KQw. *Montipora* colony 2 (*Montipora foliosa*): https://doi.org/10.5061/dryad.8kprr4xn6: https://datadryad.org/stash/share/IRhiQ_FAnFfsFCf9WyLub1q5dxFMcCsLY4zKtC0M_CQ. *Montipora* colony 3 (*Montipora turgescens*): https://doi.org/10.5061/dryad.3j9kd51js: https://datadryad.org/stash/share/1hajadPUqq1F9wVzrG14CPTZqYIGP0‐YNf05_XBuafw. *Montipora* branch 1 (*Montipora foliosa*): https://doi.org/10.5061/dryad.5hqbzkh5d: https://datadryad.org/stash/share/usOFovWkuLglp9cPoGU4uyckaVEgun41vcEkTaudF1w. *Montipora* branch 2 (*Montipora foliosa*): https://doi.org/10.5061/dryad.18931zcwt: https://datadryad.org/stash/share/sZBhqtWDIxnpZojirmcp6_DT5zYb9yP‐XpyGUWqxsBQ. *Montipora* branch 3 (*Montipora foliosa*): https://doi.org/10.5061/dryad.8pk0p2nmw: https://datadryad.org/stash/share/YB1jFYpylLH_KOswD0j1hs9l5RywsltNrDmjXktKsik. *Pocillopora* colony 1 (*Pocillopora verrucosa*): https://doi.org/10.5061/dryad.jm63xsj9h: https://datadryad.org/stash/share/dWEv9yFqzHtC5FxpOVqh7eeZ‐jsFilSEWpOcFb39NdU. *Pocillopora* colony 2 (*Pocillopora verrucosa*): https://doi.org/10.5061/dryad.9p8cz8wfs: https://datadryad.org/stash/share/xUdEDcMsE8z3dBOTIkLC48_‐vqrF4oF7xrFcbu0Go_0. *Pocillopora* colony 3 (*Pocillopora meandrina*): https://doi.org/10.5061/dryad.9zw3r22d9: https://datadryad.org/stash/share/x1s_‐‐ngY01KwiFlJ6Unca1Me6FMVpfJ9hIQY4Hsxls. *Pocillopora* branch 1 (*Pocillopora verrucosa*): https://doi.org/10.5061/dryad.9kd51c5gn: https://datadryad.org/stash/share/sHperRPPWf3_0hZaOygBBqpf_E8BmycWhas11R0lDP8. *Pocillopora* branch 2 (*Pocillopora verrucosa*): https://doi.org/10.5061/dryad.dbrv15f10: https://datadryad.org/stash/share/18HrwEDMpaucls7Ig_YAaz8DfemRbz_fRap‐YMxiXZg. *Pocillopora* branch 3 (*Pocillopora verrucosa*): https://doi.org/10.5061/dryad.zcrjdfnb5: https://datadryad.org/stash/share/NNvOs5JOstOuRTq1WxIm4g7r_4q‐LCr67ivg5f3K9QQ. *Seriatopora* colony 1 (*Seriatopora hystrix*): https://doi.org/10.5061/dryad.d7wm37q0z: https://datadryad.org/stash/share/9GaHjGV2FBcfgUWLc6imJcuXc3se8fRuAipLxePBrcc. *Seriatopora* branch 1 (*Seriatopora hystrix*): https://doi.org/10.5061/dryad.zs7h44j7w: https://datadryad.org/stash/share/RVnu8z6QvfVH0SpP7yPW9Md3O4BQAmEjgiJ0c74K9Io. *Seriatopora* branch 2 (*Seriatopora hystrix*): https://doi.org/10.5061/dryad.9zw3r22db: https://datadryad.org/stash/share/rqf2iKavForo5AikFkVLdgbaWUo‐TR5wr3qxRew9C8U. *Seriatopora* branch 3 (*Seriatopora hystrix*): https://doi.org/10.5061/dryad.kh1893255: https://datadryad.org/stash/share/NNvOs5JOstOuRTq1WxIm4g7r_4q‐LCr67ivg5f3K9QQ. *Porites* colony (*Porites lutea*): https://doi.org/10.5061/dryad.05qfttf3q: https://datadryad.org/stash/share/8KjdcXbhDj2dlT78J_PAZSP8Qaz5CcNTpFuIfnDXTUQ. *Favites* colony (*Favites speciosa*): https://doi.org/10.5061/dryad.2547d7wrb: https://datadryad.org/stash/share/37RP7WvLtYLVG0luD48MvQLFC‐m9e68ex6i630nLKhU. *Cycloseris* colony (*Cycloseris vaughani*): https://doi.org/10.5061/dryad.crjdfn34t: https://datadryad.org/stash/share/wnX6Pp‐HGVUwO0lJBmJFlKXwbRIPcl5dBlEyLZqyJF4.

## Supporting information

Figure S1

Figure S2

Figure S3

Figure S4

## Data Availability

The HRCT data that support the findings of this study are available to share. You may download the HRCT reconstruction data through following sharing links: *Acropora* colony 1 (*Acropora digitifera*): https://doi.org/10.5061/dryad.cc2fqz65g: https://datadryad.org/stash/share/JPGMRKQbQ8_87uWjAKvFYIdxZXXXS94Hm8dpDAO5Np4. *Acropora* colony 2 (*Acropora muricata*): https://doi.org/10.5061/dryad.sf7m0cg5q: https://datadryad.org/stash/share/s0FVMlF4KcOizXGbVJSBYL8TaJKUd0R0meZ4ac0f2wg. *Acropora* colony 3 (*Acropora millepora*): https://doi.org/10.5061/dryad.3r2280gfp: https://datadryad.org/stash/share/hX1oJdlviBy3ZvrDU_MHUzs3KyC4Tj4rKJnDMUtGYqU. *Acropora* branch 1 (*Acropora muricata*): https://doi.org/10.5061/dryad.bk3j9kdb8: https://datadryad.org/stash/share/8slxGygK_qcnQ2FgmSzAD5MjNjnp8U‐Gz8tnB64Ttbc. *Acropora* branch 2 (*Acropora muricata*): https://doi.org/10.5061/dryad.0cfxpnw1t: https://datadryad.org/stash/share/Hkzyc9vigW1BvdXi7xLUZNcpDl0RQ1X1NiVxErc4Axg. *Acropora* branch 3 (*Acropora muricata*): https://doi.org/10.5061/dryad.vt4b8gtrm: https://datadryad.org/stash/share/‐PxLGIBKoPLzjNqzhWX8xS1jEoEbzhwa0ixhEQPb9hc. *Montipora* colony 1 (*Montipora foliosa*): https://doi.org/10.5061/dryad.08kprr525: https://datadryad.org/stash/share/nUY3EePPILdaehpbHw4XUOccxVZ3DwSSgNSoRuV8KQw. *Montipora* colony 2 (*Montipora foliosa*): https://doi.org/10.5061/dryad.8kprr4xn6: https://datadryad.org/stash/share/IRhiQ_FAnFfsFCf9WyLub1q5dxFMcCsLY4zKtC0M_CQ. *Montipora* colony 3 (*Montipora turgescens*): https://doi.org/10.5061/dryad.3j9kd51js: https://datadryad.org/stash/share/1hajadPUqq1F9wVzrG14CPTZqYIGP0‐YNf05_XBuafw. *Montipora* branch 1 (*Montipora foliosa*): https://doi.org/10.5061/dryad.5hqbzkh5d: https://datadryad.org/stash/share/usOFovWkuLglp9cPoGU4uyckaVEgun41vcEkTaudF1w. *Montipora* branch 2 (*Montipora foliosa*): https://doi.org/10.5061/dryad.18931zcwt: https://datadryad.org/stash/share/sZBhqtWDIxnpZojirmcp6_DT5zYb9yP‐XpyGUWqxsBQ. *Montipora* branch 3 (*Montipora foliosa*): https://doi.org/10.5061/dryad.8pk0p2nmw: https://datadryad.org/stash/share/YB1jFYpylLH_KOswD0j1hs9l5RywsltNrDmjXktKsik. *Pocillopora* colony 1 (*Pocillopora verrucosa*): https://doi.org/10.5061/dryad.jm63xsj9h: https://datadryad.org/stash/share/dWEv9yFqzHtC5FxpOVqh7eeZ‐jsFilSEWpOcFb39NdU. *Pocillopora* colony 2 (*Pocillopora verrucosa*): https://doi.org/10.5061/dryad.9p8cz8wfs: https://datadryad.org/stash/share/xUdEDcMsE8z3dBOTIkLC48_‐vqrF4oF7xrFcbu0Go_0. *Pocillopora* colony 3 (*Pocillopora meandrina*): https://doi.org/10.5061/dryad.9zw3r22d9: https://datadryad.org/stash/share/x1s_‐‐ngY01KwiFlJ6Unca1Me6FMVpfJ9hIQY4Hsxls. *Pocillopora* branch 1 (*Pocillopora verrucosa*): https://doi.org/10.5061/dryad.9kd51c5gn: https://datadryad.org/stash/share/sHperRPPWf3_0hZaOygBBqpf_E8BmycWhas11R0lDP8. *Pocillopora* branch 2 (*Pocillopora verrucosa*): https://doi.org/10.5061/dryad.dbrv15f10: https://datadryad.org/stash/share/18HrwEDMpaucls7Ig_YAaz8DfemRbz_fRap‐YMxiXZg. *Pocillopora* branch 3 (*Pocillopora verrucosa*): https://doi.org/10.5061/dryad.zcrjdfnb5: https://datadryad.org/stash/share/NNvOs5JOstOuRTq1WxIm4g7r_4q‐LCr67ivg5f3K9QQ. *Seriatopora* colony 1 (*Seriatopora hystrix*): https://doi.org/10.5061/dryad.d7wm37q0z: https://datadryad.org/stash/share/9GaHjGV2FBcfgUWLc6imJcuXc3se8fRuAipLxePBrcc. *Seriatopora* branch 1 (*Seriatopora hystrix*): https://doi.org/10.5061/dryad.zs7h44j7w: https://datadryad.org/stash/share/RVnu8z6QvfVH0SpP7yPW9Md3O4BQAmEjgiJ0c74K9Io. *Seriatopora* branch 2 (*Seriatopora hystrix*): https://doi.org/10.5061/dryad.9zw3r22db: https://datadryad.org/stash/share/rqf2iKavForo5AikFkVLdgbaWUo‐TR5wr3qxRew9C8U. *Seriatopora* branch 3 (*Seriatopora hystrix*): https://doi.org/10.5061/dryad.kh1893255: https://datadryad.org/stash/share/NNvOs5JOstOuRTq1WxIm4g7r_4q‐LCr67ivg5f3K9QQ. *Porites* colony (*Porites lutea*): https://doi.org/10.5061/dryad.05qfttf3q: https://datadryad.org/stash/share/8KjdcXbhDj2dlT78J_PAZSP8Qaz5CcNTpFuIfnDXTUQ. *Favites* colony (*Favites speciosa*): https://doi.org/10.5061/dryad.2547d7wrb: https://datadryad.org/stash/share/37RP7WvLtYLVG0luD48MvQLFC‐m9e68ex6i630nLKhU. *Cycloseris* colony (*Cycloseris vaughani*): https://doi.org/10.5061/dryad.crjdfn34t: https://datadryad.org/stash/share/wnX6Pp‐HGVUwO0lJBmJFlKXwbRIPcl5dBlEyLZqyJF4.
