# Supplementary figures and images for: Micro‐CT reconstruction reveals the colony pattern regulations of four dominant reef‐building corals
Source: Ecol Evol. 2021 Nov 4;11(22):16266–79. doi: 10.1002/ece3.8308 (PMC8601894; doi:10.1002/ece3.8308)

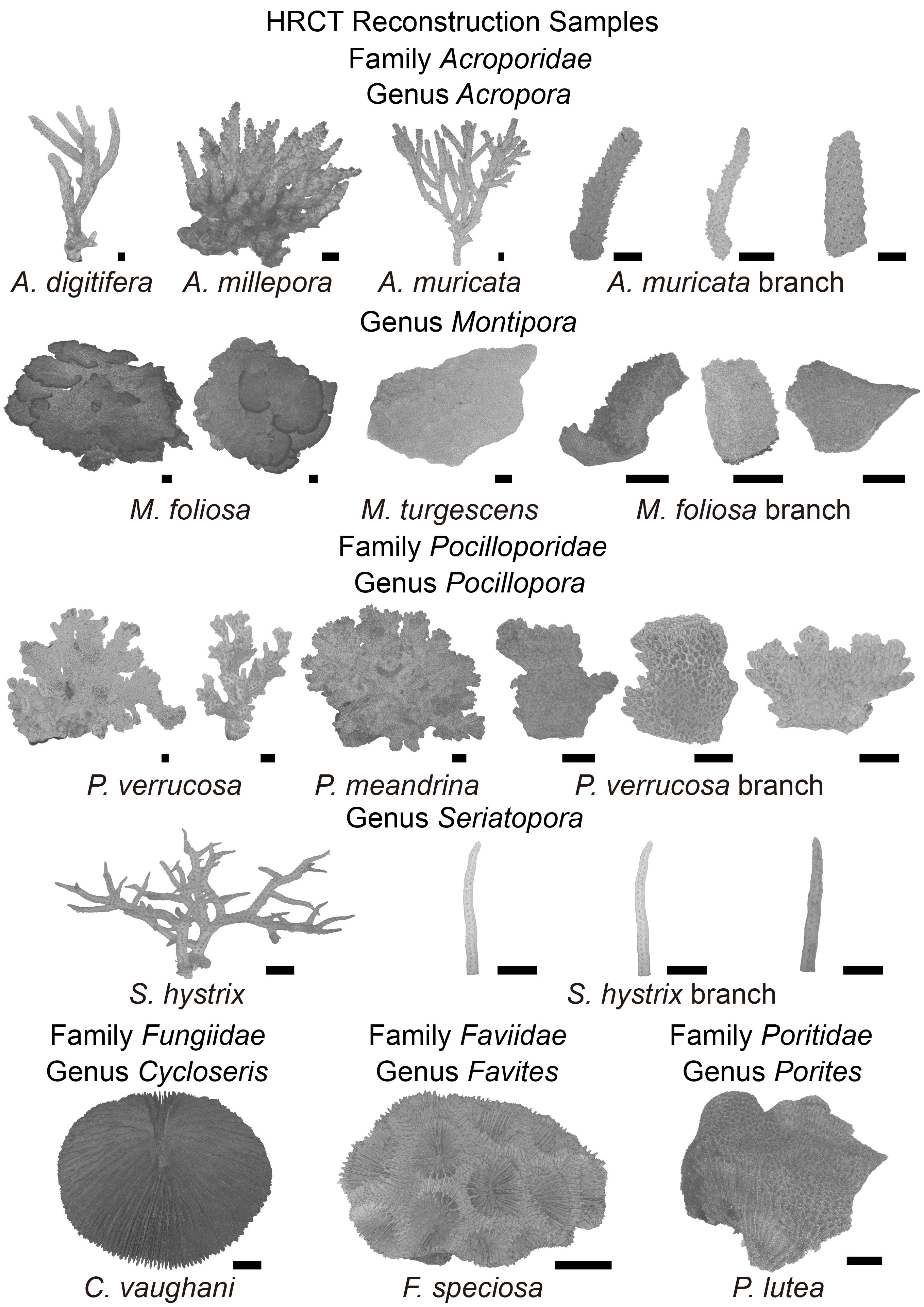

Supplement: Supplementary file 1 — Figure S1 [file ECE3-11-16266-s003.jpeg]

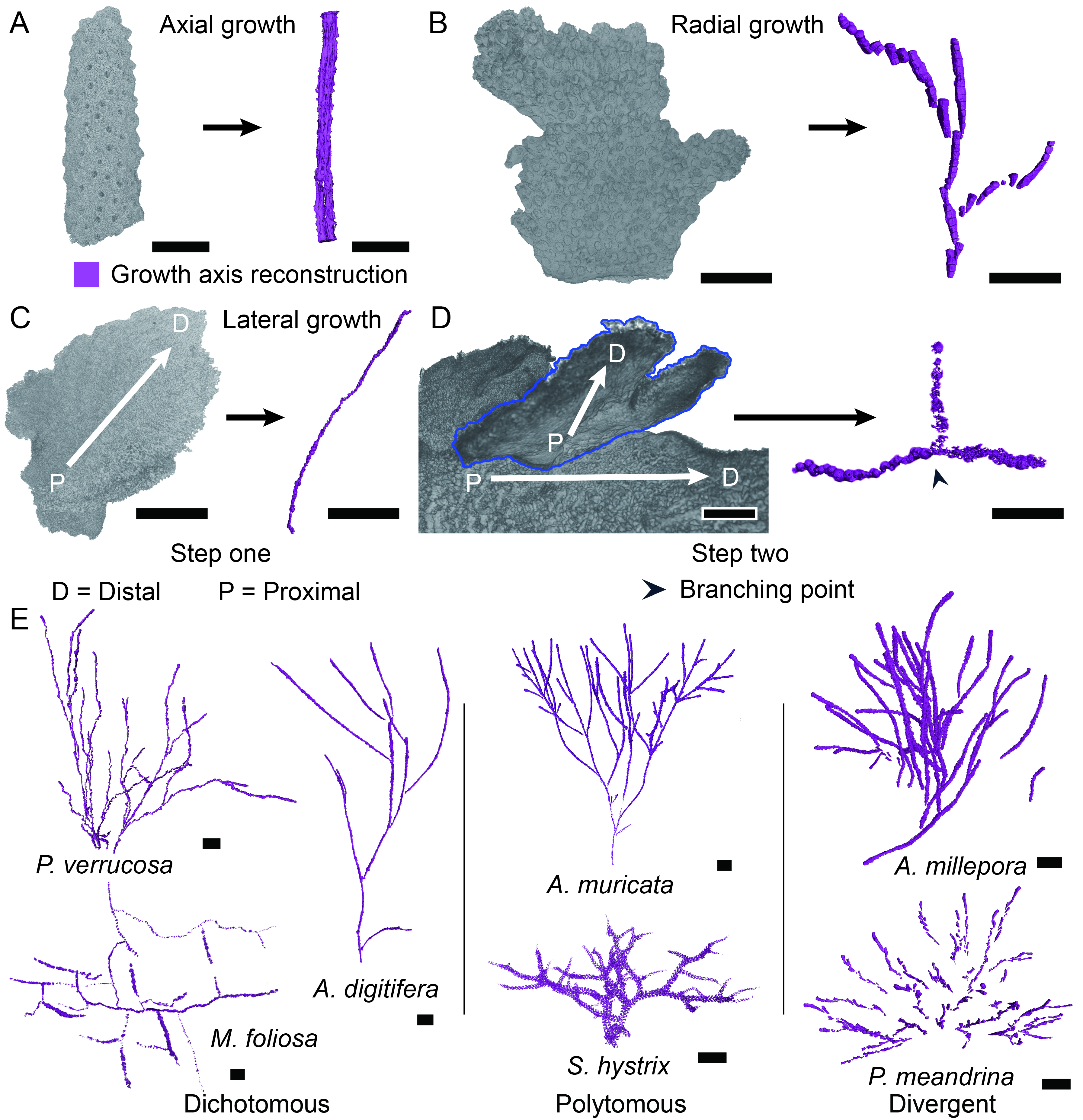

Supplement: Supplementary file 2 — Figure S2 [file ECE3-11-16266-s004.jpeg]

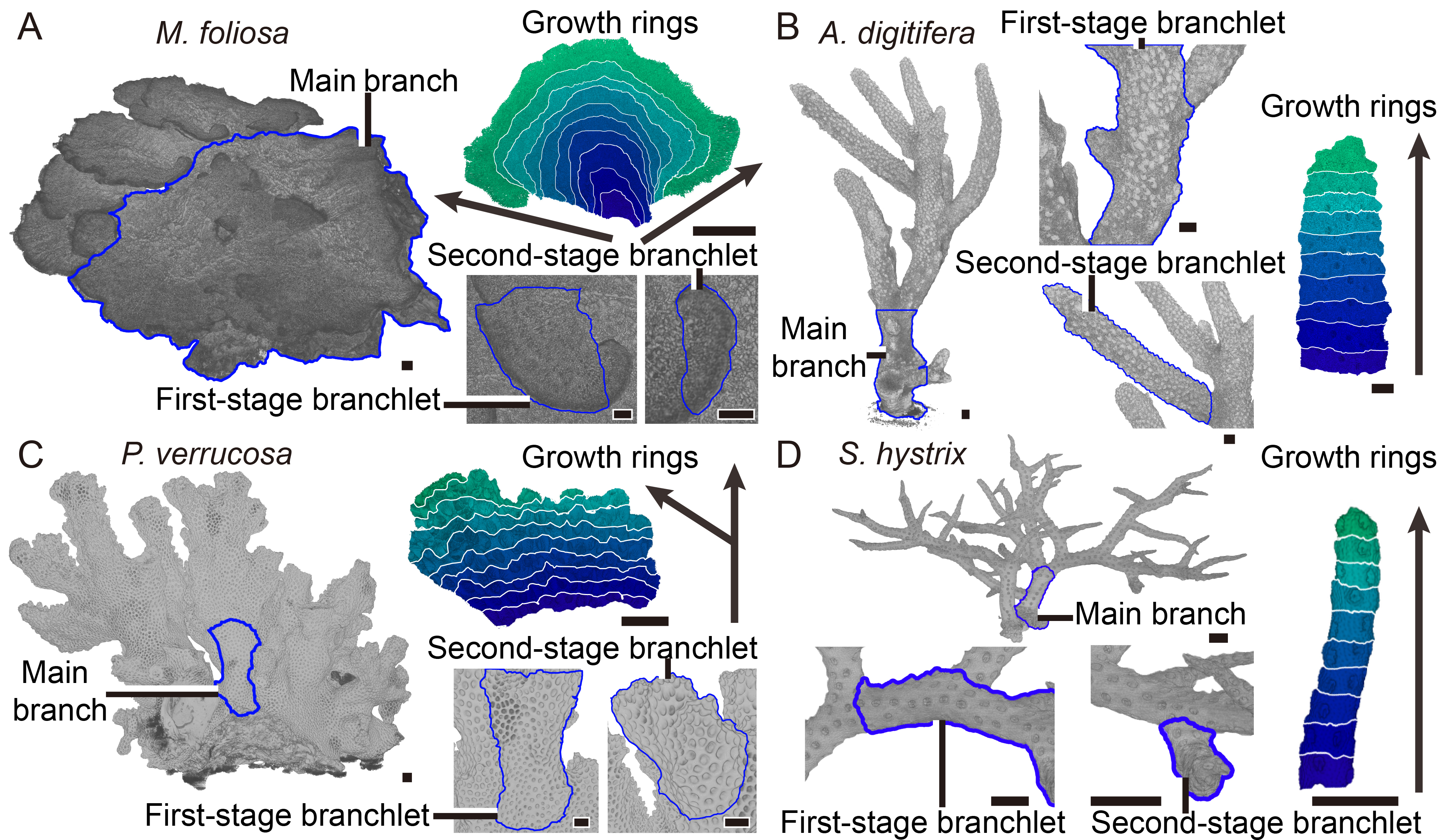

Supplement: Supplementary file 3 — Figure S3 [file ECE3-11-16266-s002.jpeg]
