# Supplementary material for: Micro‐CT reconstruction reveals the colony pattern regulations of four dominant reef‐building corals
Source: Ecol Evol. 2021 Nov 4;11(22):16266–79. doi: 10.1002/ece3.8308 (PMC8601894; doi:10.1002/ece3.8308)
Supplement: Supplementary file 4 — Figure S4 [file ECE3-11-16266-s001.docx]

**Appendix S1**

**
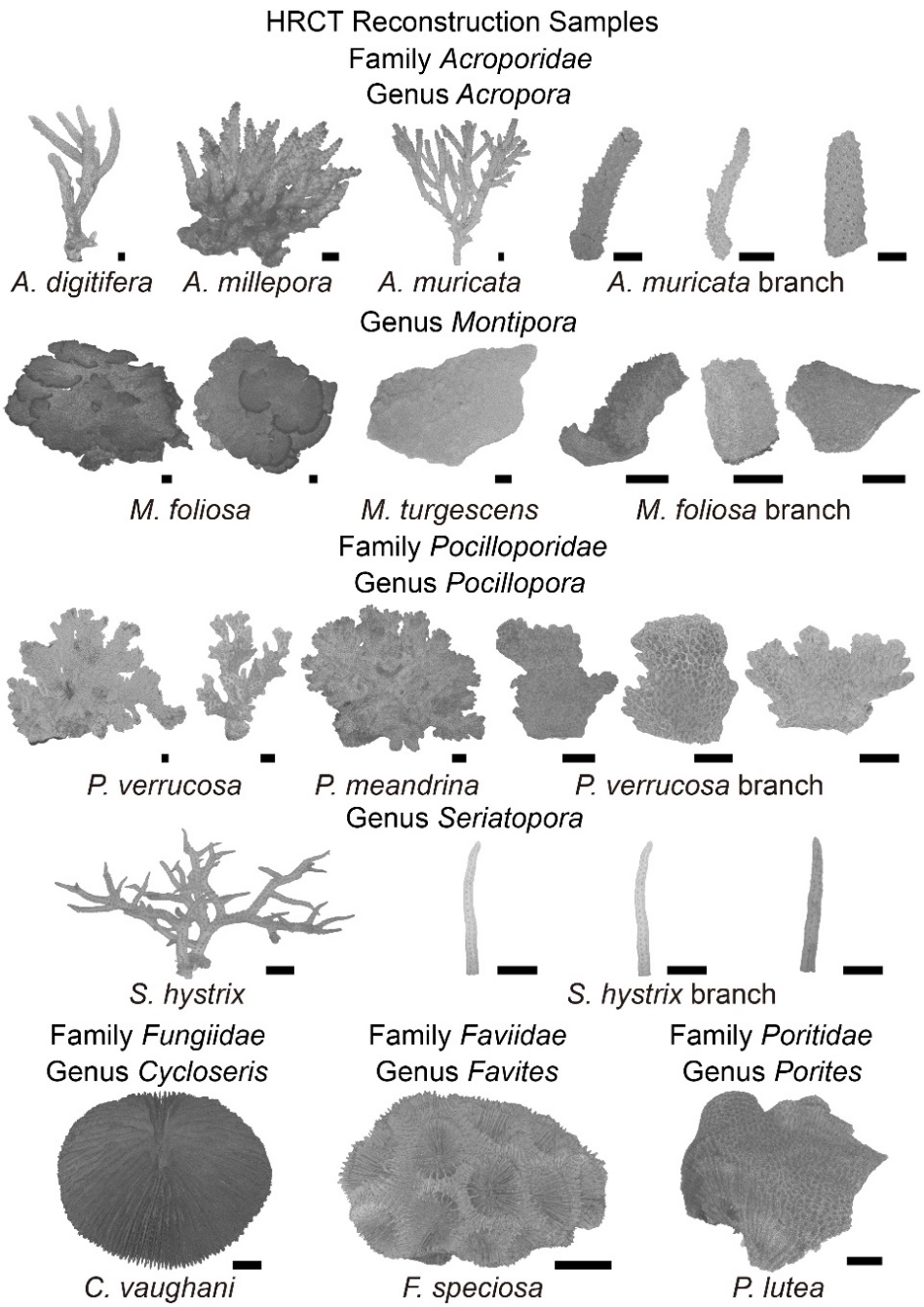
**

**Fig. S1 | Skeleton reconstructions of 25 representative colony and branch samples.**

We reconstructed 12 *Acroporidae* samples, including 6 *Acropora* samples (3 colonies and 3 branches) and 6 *Montipora* samples (3 colonies and 3 branches). There are 10 samples *Pocilloporidae* samples, including 6 *Pocillopora* samples (3 colonies and 3 branches) and 4 *Seriatopora* sample (1 colony and 3 branches). Other three samples include a *Cycloseris* colony from family *Fungiidae*, a *Favites* colony from family *Faviidae*, and a *Porites* colony from family *Poritidae*. Scale bars: 1 cm.

**
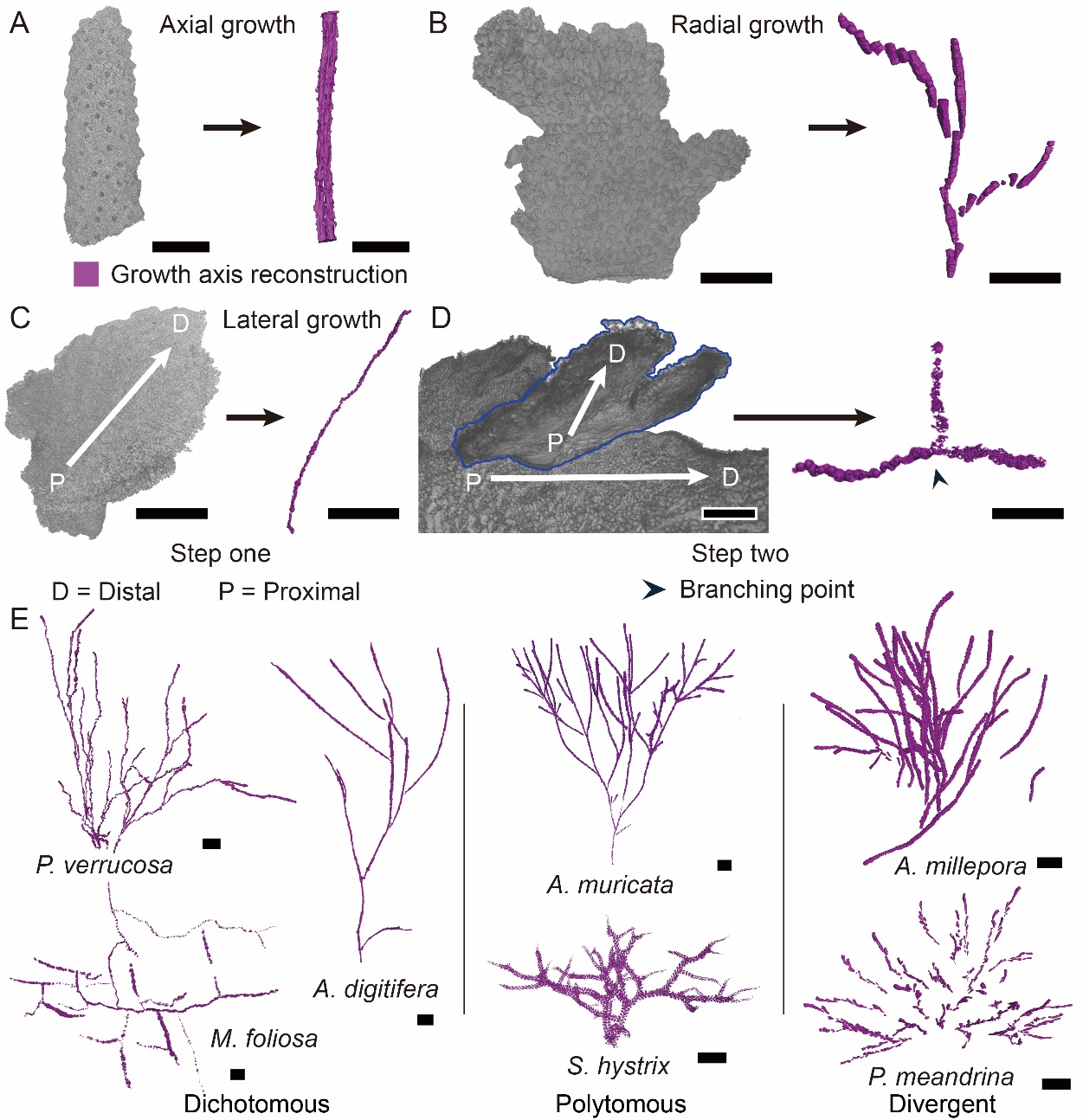
**

**Fig. S2 | The growth-axis reconstructions of axial, lateral and radial types.**

(**A**,**B**) The growth axis reconstruct methods of (**A**) axial growth and (**B**) radial growth corals. (**C**,**D**) The growth axis reconstruct methods of lateral growth corals. Step one is exploring the basic growth axis in one foliose-shape branch or branchlet from the branching point to the most distal edge by extensive canal reconstructions (55). Step two, discovering all branching points by branch growth-axis reconstructions and connecting all branch growth axis together into an entirety. (**E**) The growth axis reconstructions of 9 colony samples in 7 species can be divided into three growth categories: dichotomous, polytomous, and divergent. Scale bars: 1 cm.

**
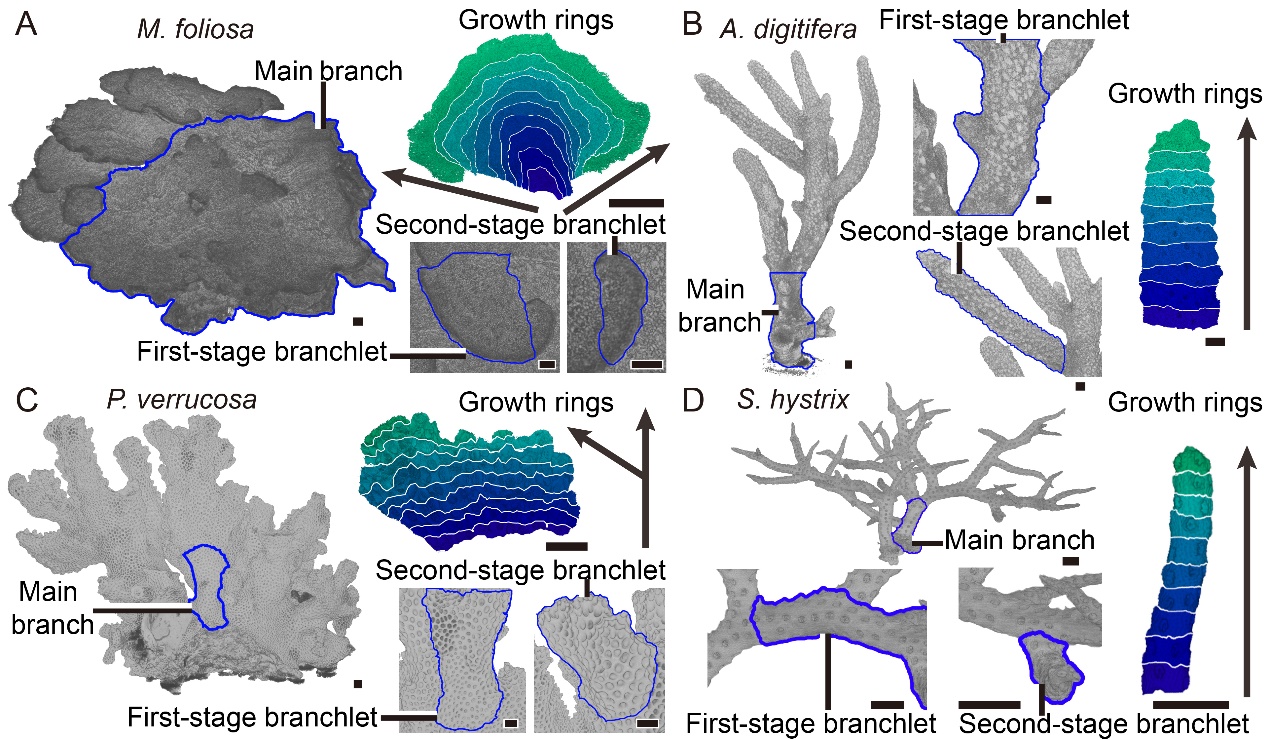
**

**Fig. S3 | The growth rings and pattern regulations of four typical coral genera.**

(**A**) *M. foliosa*, (**B**) *A. digitifera*, (**C**) *P. verrucosa* and (**D**) *S. hystrix*. Scale bars: 5 mm.

**Appendix Code | This code is used to reveal the total area of corallite and canals in each slice of the coral branch reconstructions in MATLAB.**

clear

clc

x=0:3614;

k=1;

for i=525:2600

S=['D:\MEG\nii1\','BX-D6 ',num2str(x(i),'%04d'),'.nii'];

v=spm_vol(S);

dim = v.dim;

y1=spm_read_vols(v);

y1(y1==0)=2;

y1(y1>24000)=0;

y1(y1>0)=1;

y=imdilate(y1,strel('disk',2));

y=y1;

[m1 n1]=find(y==0);

X1=length(m1);

area(k)=length(m1);

BW = im2bw(y);

IBW = ~BW;

B1 = imfill(IBW,18,'holes');

SE = ones(3);

B2 = imdilate(B1,SE,'same');

[rows,cols] = size(B2);

xx = ones(rows,1)*[1:cols];

yy = [1:rows]'*ones(1,cols);

area = sum(sum(B2));

meanx = sum(sum(double(B2).*xx))/area;

meany = sum(sum(double(B2).*yy))/area;

cen=[meanx,meany];

[m n]=find(double(B2)==1);

%k = convhull(m,n);

t2=ScatterHull([m,n],90,cen);

t2=[t2;t2(1,:)];%Closes the edges of the image through manual drawing

X2=polyarea(t2(:,1),t2(:,2));

R(k)=X1/X2;

t3=ScatterHull([m,n],90,cen);

t3=[t3;t3(1,:)];%Closes the edges of the image through manual drawing

l = sum(sqrt(diff(t3(:,1)).^2+diff(t3(:,2)).^2));

L(k)=l;

k=k+1;

clear m1 n1 m n t2 t3 X1 X2

end

save L_BX-D6 L

save Area_BX-D6 area

save R_BX-D6 R

figure;

imagesc(y');

colormap(flipud(gray));

hold on

plot(t2(:,1),t2(:,2),'r-')

axis([0 765 0 803])
